# Effect of filaggrin breakdown products on growth of and protein expression by *Staphylococcus aureus*

**DOI:** 10.1016/j.jaci.2010.09.015

**Published:** 2010-12

**Authors:** Helen Miajlovic, Padraic G. Fallon, Alan D. Irvine, Timothy J. Foster

**Affiliations:** a Moyne Institute of Preventive Medicine, Trinity College, Dublin, Ireland; b Institute of Molecular Medicine, Trinity College, Dublin, Ireland; c Department of Paediatric Dermatology, Our Lady's Children's Hospital, Crumlin, Dublin, Ireland

**Keywords:** Filaggrin, *Staphylococcus aureus*, colonization, skin, atopic dermatitis, AD, Atopic dermatitis, CA-MRSA, Community-acquired methicillin-resistant *Staphylococcus aureus*, ClfB, Clumping factor B, FnBP, Fibronectin binding protein, HRP, Horseradish peroxidase, IsdA, Iron-regulated surface determinant A, PCA, Pyrrolidone carboxylic acid, Sdr, Serine-aspartate repeat protein, Spa, Protein A, TSB, Tryptone soy broth, UCA, Urocanic acid

## Abstract

**Background:**

Colonization of the skin by *Staphylococcus aureus* in individuals with atopic dermatitis exacerbates inflammation. Atopic dermatitis is associated with loss-of-function mutations in the *filaggrin (FLG)* gene, accompanied by reduced levels of filaggrin breakdown products on the skin.

**Objective:**

To assess the affect of growth in the presence of the filaggrin breakdown products urocanic acid (UCA) and pyrrolidone carboxylic acid (PCA) on fitness of and protein expression by *S aureus*.

**Methods:**

*S aureus* was grown for 24 hours in the presence of UCA and PCA, and the density of the cultures was monitored by recording OD_600_ values. Cell wall extracts and secreted proteins of *S aureus* were isolated and analyzed by SDS-PAGE. Cell wall–associated proteins known to be involved in colonization and immune evasion including clumping factor B, fibronectin binding proteins, protein A, iron-regulated surface determinant A, and the serine-aspartate repeat proteins were examined by Western immunoblotting.

**Results:**

Acidification of growth media caused by the presence of UCA and PCA resulted in reduced growth rates and reduced final cell density of *S aureus.* At the lower pH, reduced expression of secreted and cell wall–associated proteins, including proteins involved in colonization (clumping factor B, fibronectin binding protein A) and immune evasion (protein A), was observed. Decreased expression of iron-regulated surface determinant A due to growth with filaggrin breakdown products appeared to be independent of the decreased pH.

**Conclusion:**

*S aureus* grown under mildly acidic conditions such as those observed on healthy skin expresses reduced levels of proteins that are known to be involved in immune evasion.

*Staphylococcus aureus* permanently colonizes the anterior nares of 20% of the population. Nasal carriage is a prerequisite for colonization of other sites such as skin and is a risk factor for *S aureus* infections.^1^ Host factors associated with immune responses are believed to play a role in determining carriage status.^2^ To survive on the skin, bacteria have to overcome acidic conditions as well as antimicrobial peptides and fatty acids. Colonization of the skin by *S aureus* is usually transient. However, when the skin barrier is dysfunctional, factors produced by *S aureus* can promote adhesion. Increased skin colonization with *S aureus* has been observed in patients with the chronic inflammatory skin condition atopic dermatitis (AD).^3,4^*S aureus* colonizes 5% of subjects with healthy skin, whereas *S aureus* can be isolated from lesions in 90% of adults with AD.^5,6^

Colonization of the anterior nares and skin by *S aureus* is promoted by surface-associated proteins that can bind to host adhesive molecules. Compared with healthy skin by immunohistochemical staining, higher levels of fibronectin were found in the stratum corneum of patients with AD. The expression of fibronectin binding proteins (FnBPs) A and B by *S aureus* enhanced adherence to AD skin.^7^ Other surface-associated proteins also contribute to colonization. Clumping factor B (ClfB) promotes adhesion to desquamated epithelial cells through binding cytokeratin 10.^8^*S aureus clfB* mutants were eliminated from the humans nares faster than wild-type strains, indicating the importance of this protein in nasal colonization.^9^ The serine-aspartate repeat proteins (Sdrs) C and D and the *S aureus* surface protein G also promote adhesion of bacteria to squamous cells, although their ligands are not known.^10^

The nasal mucosa and skin are iron-restricted environments that stimulate expression of iron-regulated surface determinant A (IsdA) by *S aureus*. IsdA promotes bacterial adhesion to squames through binding to loricrin, involucrin, and cytokeratin 10 on the surface of squamous cells.^11,12^ IsdA plays an important role in promoting colonization of the nares and survival of *S aureus* on skin by making the cell surface hydrophilic and conferring resistance to bactericidal lipids and cationic antimicrobial peptides.^13^ IsdA also binds lactoferrin and neutralizes its antibacterial activity.^14^

Surface-associated proteins can also contribute to inflammation associated with AD. In the respiratory tract, protein A (Spa) of *S aureus* can interact with TNF receptor 1 on the surface of airway epithelial cells to stimulate cytokine release and subsequent inflammation.^15^ Spa is believed to promote production of cytokines at skin sites in a similar manner. When used in combination with subclinical concentrations of detergent, Spa can induce AD in animal models.^16^

*S aureus* produces a variety of secreted virulence factors that exacerbate inflammatory reactions and prevent healing of skin lesions in AD. Cytolysins such as α, β, and γ toxins and Panton Valentine leukocidin are highly inflammatory. Panton Valentine leukocidin is associated with severe skin infections in previously healthy individuals caused by community-associated methicillin-resistant *S aureus* (CA-MRSA).^17^ Superantigen-production by *S aureus* strains is positively correlated with T-cell activation and increased the severity of disease in patients with AD.^18^ Exfoliative toxins disrupt epithelial barriers by cleaving desmoglein 1 in the upper epidermis.^19^ The extracellular fibrinogen-binding protein interacts with platelets and delays wound healing.^20^ Staphylokinase and the metalloprotease aureolysin inhibit the function of defensins and contribute to protection of bacteria *in vivo.*^21,22^

Recently, loss-of-function mutations in the structural protein filaggrin have been identified as a major risk factor for AD.^23-25^ Filaggrin plays an important role in epidermal barrier function. Cleavage of the precursor profilaggrin during epidermal differentiation results in 10 to 12 filaggrin peptides that promote compaction of keratinocytes into squames. Filaggrin is subsequently broken down into hygroscopic amino acids including urocanic acid (UCA) and pyrrolidone carboxylic acid (PCA). UCA and PCA are components of the natural moisturizing factor of the skin, which contributes to hydration of the stratum corneum and may also regulate pH.^26^*FLG* null mutations are associated with decreased levels of UCA and PCA on skin and impaired barrier function.^27^ Although *FLG* loss-of-function mutations are the strongest genetic risk factor for AD, the pathogenic mechanisms through which they lead to this disease remain unclear.

In this study, *S aureus* was grown in the presence of the filaggrin breakdown products UCA and PCA at concentrations similar to those found on healthy skin in human beings wild-type for *FLG*. The effects on growth rate and protein expression profile of *S aureus* strain SH1000 were assessed, in particular those surface proteins known to be involved in colonization of skin.

## Methods

### Bacterial growth conditions

Strain SH1000 was grown in complex, iron-containing tryptone soy broth (TSB) or iron- deficient RPMI at 37°C with shaking (200 rpm) Cultures were grown for 16 hours, washed, and diluted into fresh media. UCA and PCA were added to growth media at 10 mmol/L. The cell density of the cultures was monitored by recording OD_600_ values of samples taken at various time points over 24 hours.

### Preparation of solubilized cell wall proteins

Cultures of *S aureus* were washed twice in PBS and adjusted to an OD_600_ nm of 10 in 250 μL 20 mmol/L TRIS (pH 8), 10 mmol/L MgCl_2_ containing 30% raffinose (wt/vol). Complete EDTA-free protease inhibitor cocktail (Roche, Mannheim, Germany) and lysostaphin (200 μg/mL; Ambi, Lawrence, NY) were added to the cells and incubated at 37°C for 10 minutes. Protoplasts were sedimented by centrifugation at 5000 rpm for 10 minutes.^28^ A total of 10 μL of each sample supernatant containing cell wall–associated proteins was removed and analyzed by SDS-PAGE.^29^

### Preparation of secreted proteins

Cultures of *S aureus* were centrifuged, and the supernatant fraction was removed and incubated for 30 minutes at 4°C with 100% (wt/vol) trichloroacetic acid. The precipitated proteins were centrifuged and the pellet washed twice with acetone. The pellet was dissolved in PBS and analyzed by SDS-PAGE.

### Western immunoblotting

Cell wall fractions were separated on 10% (wt/vol) polyacrylamide gels, transferred onto polyvinylidene difluoride membranes (Roche), and blocked in 10% (wt/vol) skimmed milk (Marvel). Membranes were probed with polyclonal anti-ClfB, anti-IsdA, and anti-SdrB repeat antibodies (1:5000). Rabbit IgG (1:2000; Dako, Glostrup, Denmark) was used to detect Spa.

### Fibronectin affinity blot

Fibronectin-binding proteins were detected by probing membranes with biotinylated fibronectin. Human fibronectin (1 mg in 2 mL PBS) was incubated with 2 mg N-Hydroxysulfosuccinimide-biotin for 15 minutes at room temperature. The reaction was stopped by addition of 10 mmol/L NH_4_Cl. Excess biotin was removed by dialysis against PBS for 16 hours at 4°C. Biotinylated fibronectin was diluted to 30 μg/mL in TS buffer (10 mmol/L Tris-HCl, 150 mmol/L NaCl, pH 7.4) plus 5% (wt/vol) skimmed milk and incubated with PVDF membranes for 2 hours. Excess fibronectin was removed by washing with TS Tween and bound fibronectin detected by using peroxidase-conjugated streptavidin (diluted 1:2000; Roche).

### Purification of recombinant cytokeratin 10

Recombinant murine cytokeratin 10 (454-570) was purified by Ni^2+^ affinity chromatography with the addition of 6 mol/L urea (Sigma, Arklow, Ireland) to the bacterial cell suspension before lysis.^30^

### Bacterial adhesion assays

Microtiter plates (Thermo Fisher Scientific, Walldorf, Germany) were coated with serial dilutions (100 μL) of recombinant murine cytokeratin 10 (454-570) in carbonate buffer (15 mmol/L Na_2_CO_3_, 35 mmol/L NaHCO_3_, pH 9.6). Serial dilutions of fibronectin (Calbiochem, Nottingham, United Kingdom) were added to 96-well flat-bottomed plates (Sarstedt, Nümbrecht, Germany). The adhesion assays followed the same protocol after this point. After 16 hours of incubation at 4°C, the wells were washed with PBS. A total of 100 μL filtered BSA (5% wt/vol) in PBS was added to each well, and plates were incubated for 2 hours at 37°C followed by washing with PBS. Bacterial suspensions were adjusted to an OD_600_ nm of 1 and added to microtiter wells in 100 μL quantities. Plates were incubated for 2 hours at 37°C. Wells were washed, and 100 μL 25% (vol/vol) formaldehyde was added for 15 minutes to fix adherent cells. Wells were washed with PBS and stained with 100 μL 0.5% (wt/vol) crystal violet for 1 minute. Wells were washed with PBS and cell-bound crystal violet was solubilized with 100 μL 5% (vol/vol) acetic acid.^31^ The absorbance at 570 nm (A_570nm_) was determined with an ELISA plate reader (Labsystems).

## Results

### Growth of *S aureus* in the presence of UCA and PCA

**Fig 1:**
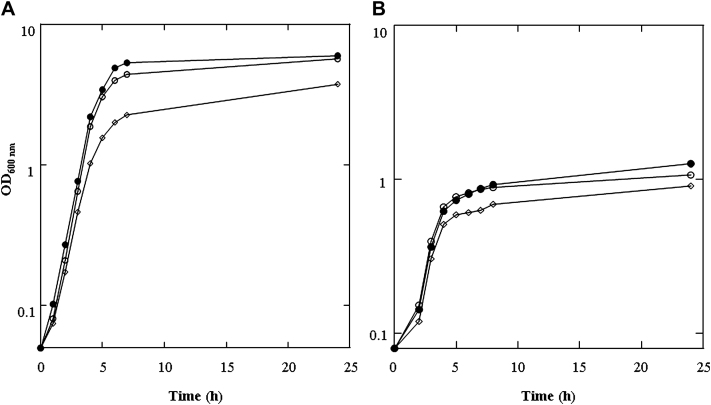
Effect of filaggrin breakdown products on growth of *S aureus*. Strain SH1000 was grown in TSB (**A;** ●) and RPMI (**B;** ●). UCA and PCA, 10 mmol/L (◊), were added to the growth media and to a control culture in which pH was neutralized after addition of UCA and PCA (○). Cell density of cultures was recorded at the indicated time points.

*S aureus* strain SH1000 was used in this study because it expresses the majority of surface-associated proteins involved in colonization. SH1000 was grown in a complex iron-containing medium, TSB, or in RPMI, which is deficient in iron and other nutrients, reflecting conditions present on skin. UCA and PCA (10 mmol/L) were added to the bacterial growth medium, mimicking the reported concentrations of 6 to 12 mmol/L found on healthy skin.^32^ The addition of UCA and PCA reduced the neutral pH of TSB and RPMI to 5.4 and 5.5, respectively. As a control, SH1000 was also grown in TSB or RPMI in which the pH was adjusted to 7 with NaOH after addition of UCA and PCA. The cell density of the cultures was monitored by recording the OD_600_ values. The doubling time of SH1000 growing in TSB was 39 minutes, and the final yield of bacterial cells after growth for 24 hours was an OD_600 nm_ of 6 (Fig 1, *A*). Growth in the presence of UCA and PCA resulted in an increased doubling time (49 minutes), and the final yield of cells was reduced by 38% (OD_600 nm_, 3.7.) Neutralizing the pH after addition of UCA and PCA resulted in growth rates (doubling time of 41 minutes) and cell densities (OD_600 nm_, 5.7) similar to those seen when SH1000 was grown in TSB.

Similar results were obtained when SH1000 was grown in RPMI. The doubling time was extended in the presence of UCA and PCA from 59 to 63 minutes and the final cell density reduced by 30% (from OD_600 nm_ of 1.27 to 0.91). Adjusting the pH to 7 after addition of UCA and PCA resulted in growth rates (doubling time of 59 minutes) and cell densities (OD_600 nm_, 1.1) similar to those seen when grown in RPMI alone (Fig 1, *B*). Therefore, the extended growth rate and reduced cell density of SH1000 grown in both TSB and RPMI appeared to be pH-dependent rather than a direct effect of the filaggrin breakdown products. To confirm this, SH1000 was grown in TSB adjusted to pH 5.5 with HCl. This resulted in a decrease in final cell density and increased doubling times compared with cells grown at pH 7 (see this article's Fig E1 in the Online Repository at www.jacionline.org).

The experiments described were performed 3 times with similar results.

### Expression of cell wall associated proteins and secreted proteins

Total protein expression by *S aureus* grown in the presence of UCA and PCA both individually and together was assessed. Proteins isolated from the cell walls and supernatant fractions were separated by SDS-PAGE and visualized by staining with Coomassie blue.

**Fig 2:**
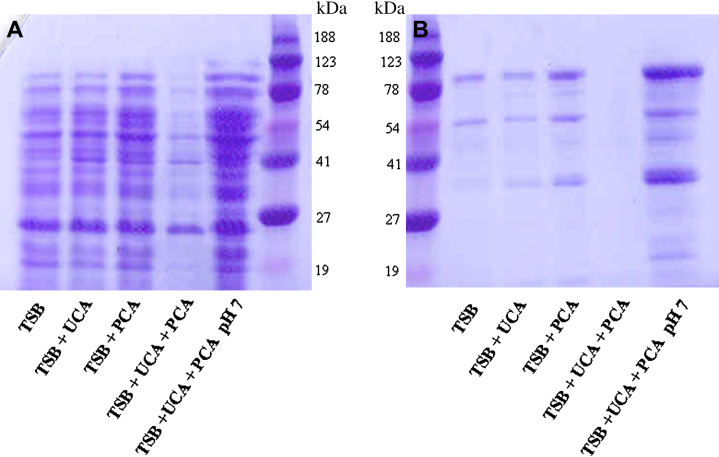
Effect of filaggrin breakdown products on protein expression by *S aureus*. Strain SH1000 was grown in TSB, TSB supplemented with 10 mmol/L UCA and/or PCA, and TSB in which pH was neutralized after addition of UCA and PCA. Cell wall **(A)** and secreted protein fractions **(B)** were separated on 10% SDS-PAGE gels and visualized by Coomassie staining.

The addition of 10 mmol/L UCA or PCA to TSB reduced the pH from 7.2 to 6.0 and 6.5, respectively. No major differences were seen in the profiles of cell wall–associated proteins when UCA or PCA was added to TSB individually. The addition of both filaggrin breakdown products to TSB reduced the pH to 5.4. This resulted in a dramatic decrease in surface-associated proteins expressed by SH1000. The effect could be alleviated by neutralizing the pH, indicating that the reduction in expression was pH-dependent and not a direct effect of the filaggrin breakdown products (Fig 2, *A*). A similar effect was seen with proteins secreted by SH1000, which were decreased in a pH-dependent manner in the presence of both UCA and PCA (Fig 2, *B*).

Cell wall proteins and secreted proteins were isolated 3 separate times and the experiments described repeated with each of these samples with similar results.

### Expression of surface-associated proteins involved in colonization and inflammation of skin

Most surface-associated proteins are expressed predominantly in the exponential phase of growth. SH1000 was grown in TSB to the midexponential (OD_600 nm_, 0.45) and stationary (OD_600 nm_, 6) phases, and cell wall–associated proteins were solubilized and analyzed by Western immunoblotting. Membranes were probed with antibodies specific for ClfB and the B repeat regions of the Sdr proteins. FnBPs were detected by incubating membranes with biotin-labeled fibronectin. Spa was detected with horseradish peroxidase (HRP)–labeled rabbit IgG.

**Fig 3:**
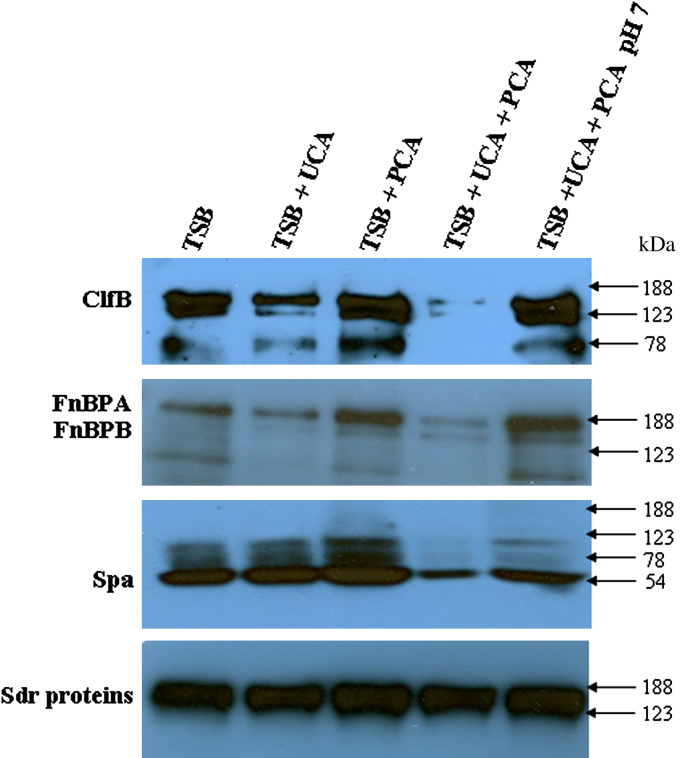
Expression of proteins involved in skin colonization and inflammation. SH1000 was grown in TSB, TSB supplemented with 10 mmol/L UCA and/or PCA, and TSB in which pH was neutralized after addition of UCA and PCA. Cell wall proteins were isolated, separated by SDS-PAGE, and electroblotted onto PVDF membranes. Membranes were probed with anti-ClfB antibody, biotin-labeled fibronectin, HRP-conjugated IgG, and anti-SdrB repeat antibody.

Growth of SH1000 in the presence of 10 mmol/L PCA had no significant effect on expression of ClfB (150 kd), FnBPA and FnBPB (180 and 150 kd, respectively), Spa (54 kd), or the Sdr proteins (150-180 kd). Growth in the presence of 10 mmol/L UCA reduced expression of ClfB and FnBPA to a small degree. When both UCA and PCA were added to the growth medium, a dramatic reduction in the levels of ClfB, FnBPA, and Spa was seen. This appeared to be an effect of reduced pH of the growth medium because expression of these proteins could be restored by adjusting the pH to 7. To confirm this, the pH of TSB was reduced to 5.5 by using HCl, which resulted in decreased expression of ClfB, FnBPA, and Spa (see this article's Fig E2 in the Online Repository at www.jacionline.org). Expression of Sdr proteins by SH1000 was not affected by growth in the presence of UCA and PCA (Fig 3).

**Fig 4:**
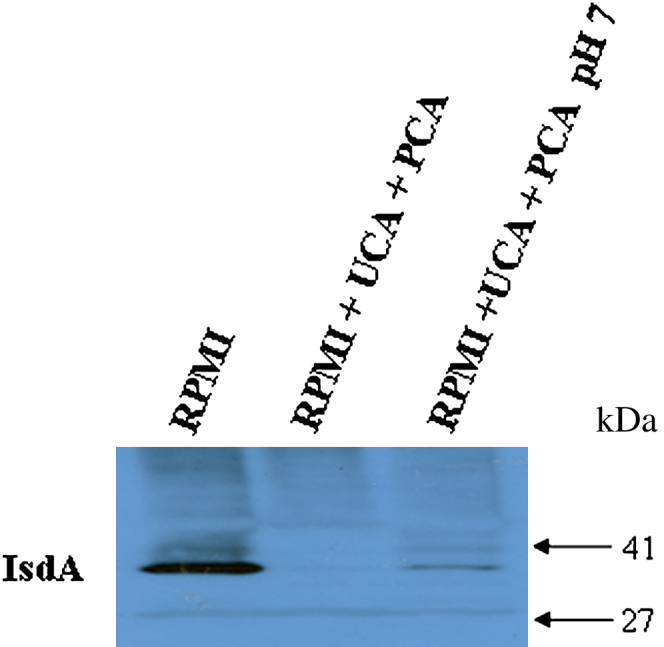
Effect of filaggrin breakdown products on expression of IsdA by *S aureus*. SH1000 was grown in RPMI, RPMI supplemented with 10 mmol/L UCA and PCA, and RPMI in which pH was neutralized after addition of UCA and PCA. Cell wall proteins were isolated, separated by SDS-PAGE, and electroblotted onto PVDF membranes. Membranes were probed with anti-IsdA antibody.

Iron-regulated surface determinant A is expressed only under iron-limiting conditions and is known to increase survival of *S aureus* on skin. SH1000 was grown in RPMI to promote expression of IsdA. In the presence of 10 mmol/L UCA and PCA, the 38-kd IsdA protein could not be detected. Expression was increased to a small degree by adjusting pH to 7 after addition of the filaggrin breakdown products. However, expression was still significantly lower than when grown in RPMI alone (Fig 4). This indicates that UCA and PCA may have a direct effect on IsdA expression.

Cell wall proteins and secreted proteins were isolated 3 separate times and the experiments described repeated with each of these samples with similar results.

### Functionality of surface-associated proteins

Clumping factor B promotes adhesion of *S aureus* to cornified epithelial cells by binding to cytokeratin 10. Increased levels of fibronectin are present in skin of patients with AD and might contribute to enhanced bacterial colonization. Bacterial adhesion assays were carried out to determine whether the reduced levels of adhesins expressed in the presence of UCA and PCA correlated with reduced binding to cytokeratin 10 and fibronectin.

**Fig 5:**
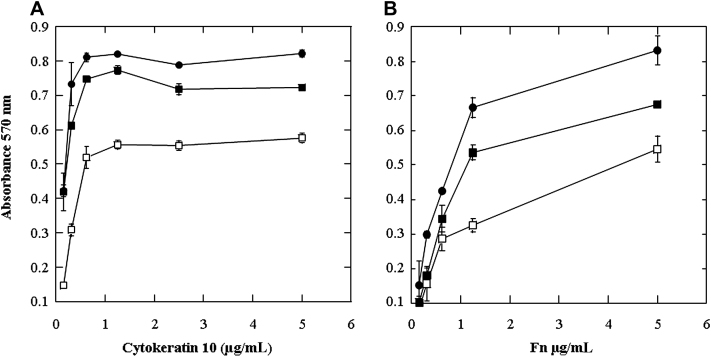
Adherence of *S aureus* to host molecules. SH1000 was grown in TSB (●), TSB supplemented with 10 mmol/L UCA and PCA (□), and TSB in which pH was neutralized after addition of UCA and PCA (■). SH1000 cultures adjusted to OD_600_ of 1.0 were added to cytokeratin 10–coated **(A)** and fibronectin *(Fn)*–coated **(B)** microtiter plates. After incubation at 37°C, adherent cells were detected by crystal violet staining.

SH1000 was grown to the exponential phase in the presence of UCA and PCA. Bacterial cells were washed, and adherence to immobilized cytokeratin 10 was measured. SH1000 grown in TSB with UCA and PCA adhered to cytokeratin 10 at significantly lower levels than when grown in TSB alone. Adherence was increased by adjusting the pH of the growth medium to 7 after addition of UCA and PCA (Fig 5, *A*). Adherence to fibronectin was also reduced by growth of SH1000 in the presence of UCA and PCA. Although adjusting the pH to 7 did increase binding to fibronectin to some degree, it was lower than when strains were grown in TSB alone (Fig 5, *B*). These results indicate that adherence of SH1000 to host molecules is reduced by growth in the presence of filaggrin breakdown products because of reduction in pH. Reduced adherence to cytokeratin 10 and fibronectin was also observed when SH1000 was grown in TSB adjusted to pH 5.5 with HCl (see this article's Fig E3 in the Online Repository at www.jacionline.org).

The adherence values in Fig 5, *A and B,* represent the means ± SDs of triplicate wells. The experiments were performed 3 times with similar results.

## Discussion

It is well established that patients with AD are colonized extensively with *S aureus*. The mechanisms by which this bacterium is able to colonize AD skin are not fully understood. Several factors are likely to contribute. Patients with AD have higher levels of T_H_2 cytokines that are believed to inhibit expression and function of antimicrobial peptides.^33^ Increased levels of fibronectin in the stratum corneum of patients with AD may allow increased adhesion through fibronectin-binding proteins.^7^ The increased pH of atopic skin compared with normal skin may also create more favorable growth conditions for *S aureus*.

The pH of skin is crucial in maintaining the epidermal barrier function, desquamation, and lipid synthesis and as an important defense mechanism against pathogens.^34^ The pH of healthy skin ranges from 4 to 6. It is maintained by the fatty acids found in sweat and sebum secretions, by products of phospholipid hydrolysis, and by sodium-proton ion exchangers of lamellar bodies.^34,35^ Filaggrin breakdown products that make up natural moisturizing factor also play a role in maintaining the acidic pH of skin.^36^ In AD, the pH of skin shifts toward alkalinity, and a positive correlation has been found between higher pH and the severity of the disease.^37^ Several factors contribute to increased pH in AD skin, including decreased sweat secretion and decreased levels of free fatty acids. Null mutations in the *FLG* gene result in reduced levels of UCA and PCA, which may contribute to increased skin pH in a proportion of patients with AD.^26,34^*In vitro* studies have shown the T_H_2 cytokines IL-4 and IL-13 act synergistically in reducing *FLG* expression so that these breakdown products may be reduced in active AD lesions even in individuals wild-type for *FLG*.^38^

*S aureus* can grow and divide over a pH range from 5 to 9. However, growth is inhibited at the extremes of the range.^34,39^ These previous observations are supported by the results of this study. *S aureus* strain SH1000 grown in rich media and under iron-limiting conditions had slower doubling times and decreased final yields when grown in the presence of acidic filaggrin breakdown products. UCA and PCA at 10 mmol/L decreased the pH of neutral TSB and RPMI to 5.4 and 5.5, respectively. These values are comparable to the pH of healthy skin. Growth was restored by adjusting the pH of the growth medium to neutral after addition of UCA and PCA (Fig 1, *A and B*).

The impact of mild acid on protein expression by *S aureus* has been assessed in previous studies. Weinrick et al^39^ compared expression of genes by *S aureus* at pH 5.5 and 7.5. They identified over 400 genes in which transcription was affected by low pH and classed them as belonging to the mild acid stimulon. Many of their observations are supported by the results of this study, including decreased expression of some secreted proteins and of the surface-associated proteins Spa and FnBPA at pH 5.5. Weinrick et al^39^ postulated that the effect on secreted proteins was a result of downregulation of the locus for *S aureus* exoprotein expression (*sae*) at low pH. The 2 component regulatory system SaeRS encoded by the *sae* locus also affects expression of the surface-associated proteins Spa and the FnBPs. Downregulation of the regulator of toxins at pH 5.5 may explain the reduction in ClfB expression seen in this study, because regulator of toxins positively regulates ClfB expression. Growth in the presence of filaggrin breakdown products at stratum corneum concentrations resulted in a decrease in pH that reduced expression of secreted proteins and surface-associated proteins including ClfB, FnBPA, and Spa. *S aureus* proteins ClfB and FnBPA promote colonization by binding to host molecules cytokeratin 10 and fibronectin. Adherence to cytokeratin 10 and fibronectin was reduced in a pH-dependent manner correlating with the reduced expression of these proteins (Fig 5, *A and B*). Reduction of *S aureus* adhesion to keratinocytes at low pH has previously been reported.^40^ The increased alkalinity of skin in AD may allow increased expression of proteins such as ClfB and FnBPA by *S aureus* and promote colonization. Subsequent inflammation may be induced through increased expression of Spa and secreted exotoxins. In addition to the effect on growth and protein expression, acidic pH affects the 3-dimensional structure of *S aureus* enterotoxins.^41^

Expression of IsdA, a protein that promotes adhesion to squamous cells and also enhances survival on human skin, seemed to be directly affected by the presence of UCA and PCA. Unlike the other proteins studied here, expression was not restored by neutralizing the pH after addition of the filaggrin breakdown products (Fig 4). Expression of IsdA makes the surface of *S aureus* more hydrophilic, increasing resistance to lipids and cationic antimicrobial peptides found on skin.^13^ A reduction in filaggrin breakdown products on AD skin, either because of *FLG* null alleles or secondary to a T_H_2 inflammation–driven decrease in *FLG* expression, may increase expression of staphylococcal IsdA and could promote increased survival of *S aureus.* Fatty acids found on skin such as oleic acid and antimicrobial peptides such as dermicidin I are more effective at killing *S aureus* at pH 5 than at neutral pH.

Some strains of *S aureus* have acquired a mechanism to overcome the inhibitory effects of skin pH. Recently CA-MRSA strains have emerged that cause severe skin infections in previously healthy individuals. CA-MRSA strain USA-300 has an arginine catabolic mobile element that encodes enzymes of the arginine deiminase pathway, which convert arginine into carbon dioxide, ATP, and ammonia. It has been suggested that production of ammonia neutralizes the acidic environment of the skin and may be responsible for the rapid spread of CA-MRSA.^42^

Because of frequent treatment for *S aureus* infections, patients with AD are susceptible to colonization with methicillin-resistant *S aureus*. Another complication results from the development of steroid-resistant AD because of colonization with *S aureus* strains producing high levels of superantigens.^43,44^ Treatments which decrease the pH of AD skin can be effective in reducing the severity of disease by preventing colonization with *S aureus* and improving epidermal function.^6,45^ Application of low-pH creams and acidic electrolytic water on epithelial surfaces have been used effectively in previous studies to reduce colonization by *S aureus* and the severity of dermatitis.^46,47^

Here we have shown that the principal filaggrin breakdown products UCA and PCA, known to be significantly reduced in *FLG* null mutation carriers, exert profound effects on *S aureus* at physiological concentrations. The effects on staphylococcal proliferation, adhesion, and survival are largely pH-dependent but they appear to have a direct effect on IsdA expression. Although these are *in vitro* studies, they do suggest additional pathomechanisms through which carriers of *FLG* null alleles or patients with AD with reduced *FLG* expression may have enhanced susceptibility to *S aureus* colonization. These data suggest a mechanistic link between the strongest genetic factors for AD and the strongest microbial influence on the disease.

**Key messages**

• Growth of *S aureus* in the presence of physiological concentrations of filaggrin breakdown products UCA and PCA resulted in extended doubling times and decreased final yields of cells.
• *S aureus* grown in the presence of UCA and PCA had decreased levels of protein expression, including proteins involved in colonization and immune evasion.
• The effect of UCA and PCA on growth and protein expression was pH-dependent and could be alleviated by neutralizing the pH, except in the case of IsdA.

## Figures and Tables

**Figure:**
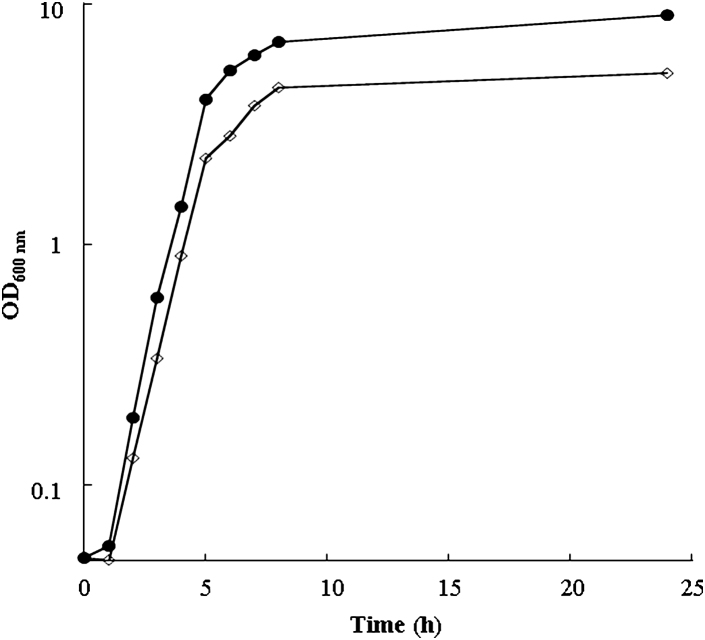
Effect of acid on growth of *S aureus*. Strain SH1000 was grown in TSB (pH 7; ●) and TSB adjusted to pH 5.5 (◊). Cell density of cultures was recorded at the indicated time points.

**Figure:**
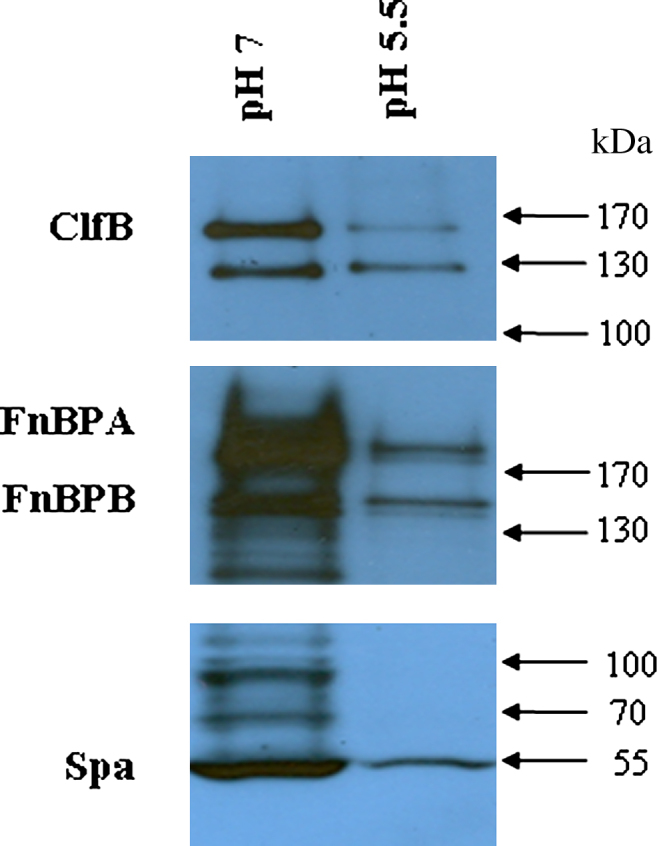
Expression of proteins involved in skin colonization and inflammation. SH1000 was grown in TSB (pH 7) and TSB adjusted to pH 5.5. Cell wall proteins were isolated, separated by SDS-PAGE, and electroblotted onto PVDF membranes. Membranes were probed with anti-ClfB antibody, biotin-labeled fibronectin, and HRP-conjugated IgG.

**Figure:**
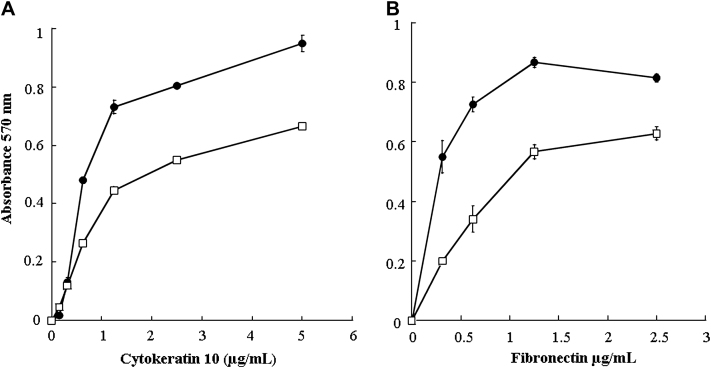
Adherence of *S aureus* to cytokeratin 10 and fibronectin. SH1000 was grown in TSB (pH 7 ●) and TSB adjusted to pH 5.5 (□). SH1000 cultures were adjusted to OD_600_ of 1.0 and added to cytokeratin 10–coated **(A)** and fibronectin-coated **(B)** microtiter plates. After incubation at 37°C, adherent cells were detected by crystal violet staining.
